# An Exploratory Feasibility Study of Incorporating Volunteering Into Treatment for Adolescent Depression and Anxiety

**DOI:** 10.3389/fpsyg.2022.840881

**Published:** 2022-04-29

**Authors:** Parissa J. Ballard, Stephanie S. Daniel, Grace Anderson, Aubry N. Koehler, Elimarie Caballero Quinones, Ashley Strahley, Linda M. Nicolotti

**Affiliations:** ^1^Department of Family and Community Medicine, Wake Forest School of Medicine, Winston-Salem, NC, United States; ^2^Department of Psychology, Wake Forest University, Winston-Salem, NC, United States; ^3^Pediatric Psychology and Behavioral Health, Pediatric Gastroenterology, Wake Forest School of Medicine, Winston-Salem, NC, United States; ^4^Wake Forest Clinical and Translational Science Institute, Winston-Salem, NC, United States

**Keywords:** affective disorders, adolescents, volunteering, mental health, treatment

## Abstract

Community volunteering is an under-utilized, at least under-researched, strategy to supplement existing treatment for affective disorders. We present findings from a feasibility study incorporating community volunteering into clinical treatment for depression and anxiety among adolescents and young adults. This exploratory pilot study had four aims: to investigate recruitment feasibility; to describe participants’ experiences with volunteering; to explore psychosocial assets by which volunteering might decrease depressive and anxiety symptoms; and to document preliminary changes in mental health outcomes before and after the volunteering intervention. Interviews and surveys were employed with participants (*N* = 9; ages 14–20, Mage = 16 years old; eight women and one man) newly diagnosed with: mild to moderate depression (single episode), mild to moderate anxiety, or adjustment disorder. Recruitment was feasible overall, successes and challenges are discussed. Experiences with volunteering were very positive. Qualitative findings revealed perceived positive effects of volunteering on mood and well-being such as helping with social anxiety and being a positive distraction. Qualitative findings revealed several psychosocial assets that improved related to volunteering (e.g., sense of purpose/meaning and sense of community). On average, participants reported a 19% decrease in depressive symptoms from the pre-survey (before volunteering) to the post-survey (after volunteering). Although more research is warranted, the implication of this study for practicing psychologists treating adolescents and young adults for mild to moderate depression and/or anxiety is that they may wish to consider incorporating community volunteer activities into treatment. Volunteering was a desirable activity for interested participants in treatment for affective disorders.

## Introduction

Community volunteering, or taking part in unpaid work for the benefit of others, can present young people with a transformative opportunity to develop positive relationships, social connections, and skills while contributing to their community. Research investigating associations between volunteering, mental health and well-being points to robust positive associations [e.g., Piliavin and Siegl (2015)] using observational methods [e.g., Jenkinson et al. (2013)] with community samples. Volunteering has not yet been systematically examined as a potential supplement or adjunct to treatment interventions with adolescents and young adults in clinical treatment for affective and/or anxiety disorders.

Affective disorders, such as depression and anxiety, are salient mental health concerns during adolescence and young adulthood [e.g., Beesdo et al. (2009)] and effective treatment interventions to address affective disorders are highly significant. Effective pharmacological and non-pharmacological treatment approaches (such as cognitive behavioral therapy) for depression (Butler et al., 2006; Weersing et al., 2017) and anxiety (Davis et al., 2011) are well-established, and innovations consistently improve and supplement treatment approaches. Non-pharmacological interventions, such as those based in positive psychology, are desirable to many people either instead of or in conjunction with medication. Based on theoretical and empirical findings detailing positive associations between volunteering and mental health and well-being (Konrath and Brown, 2013; Piliavin and Siegl, 2015; Creaven et al., 2017; Ballard et al., 2021), we believe that volunteering can help young people develop psychosocial assets and reduce symptoms of affective disorders. Importantly, volunteering fits within existing evidenced-based approaches to treating depressive and/or anxiety disorders [see Ballard et al. (2021) for full review] and is conceptually consistent with principles of positive psychology interventions, cognitive-behavioral therapy (CBT) and behavioral therapy approaches, including exposure therapy for certain anxiety disorders (Reinecke et al., 1998; Heimberg and Becker, 2002; Butler et al., 2006; David-Ferdon and Kaslow, 2008). Volunteering may also be a form of behavioral activation. In addition, it may reduce symptoms common to both depression and anxiety like rumination and overthinking by helping teens broaden their perspectives and potentially shift their thought patterns and orientation from self-focus to include a focus on others, consistent with CBT strategies. Volunteering can also provide a structured, predictable, and supportive social environment to make connections with others in a task-oriented way with an accountability structure, which provides a mechanism for socialization that may be a helpful example of “*in vivo*” social exposure in an exposure therapy approach for social anxiety (Rodebaugh et al., 2004). Therefore, community volunteerism may be a promising but under-utilized, or at least under-researched, strategy to supplement clinical treatment for adolescent affective disorders.

This pilot study incorporated community volunteering into clinical treatment for adolescent depression and anxiety to examine feasibility and collect qualitative data to explore participant experiences. First, we investigate the feasibility of recruiting participants from community behavioral health practices into the study. Next, we describe participants’ experiences with volunteering and explore psychosocial assets by which volunteering might decrease depressive and anxiety symptoms. Finally, we document preliminary changes in mental health outcomes before and after the volunteering intervention.

## Materials and Methods

### Participants

Participants were adolescents and young adults (*N* = 9; between ages 14–20, Table 1). Eligible participants were newly diagnosed (treatment <8 months) with mild to moderate depression or anxiety (single episode), comorbid depression and anxiety, or an adjustment disorder. Inclusion criteria also included elements specific to participating in an fMRI scan such as: being right-handed (which can affect brain functioning on certain tasks), not experiencing claustrophobia, and no contraindications for an fMRI scan.^1^

**TABLE 1:** Description of the sample (*N* = 9).

Sample descriptives	
**Gender**	
Female	8
Male	1
**Age**	
14	2
15	3
16	0
17	1
18	1
19	2
**Current education**	
High school	7
College	2
**SES**	
Middle income	5
Middle/upper income	3
Upper income	1
**Community**	
Urban	1
Rural	2
Suburban	6
**Race**	
White	7
Black/African American	2
**Diagnoses**	
MDD (single episode)	3
Anxiety (mild/moderate)	2
MDD and anxiety	2
Adjustment disorder	2

### Measures

*Depression* was measured through the Beck Depression Inventory-II [BDI-II; Beck et al. (1996)]. Each of the 21 items is answered on a scale from 0–3 and items were summed. Scores of 0–13 are considered in the *minimal* range; 14–19 as *mild*; 20–28 as *moderate*, and 29–63 as *severe* depressive symptoms. *Anxiety* was examined using four relevant items from the BDI that are central to a generalized anxiety disorder diagnoses according to the DSM-V (agitation, irritation, changes in sleep patterns, and concentration difficulties). This study did not include an explicit measure of anxiety; when it emerged as an important construct for study inclusion and in the qualitative data, we used relevant items from the BDI to examine indicators of anxiety in the absence of a validated full measure.

### Procedure

With appropriate ethical and IRB approvals (IRB00053435), participants were screened by community mental health providers and referred to the study team, who explained study details and obtained written consent/assent from participants (and their parents for minors). The research team administered the pre-self-report survey and scheduled a pre- fMRI lab visit before the volunteering. We helped participants find a meaningful volunteer experience from a menu of local options, developed by the research team in a series of steps (contact first author for details) to support personal meaning and goodness-of-fit with volunteering experience. Participants were asked to report their volunteer hours weekly, and we checked in with participants every 2–4 weeks via text about the volunteering. When participants had completed 30 h, we conducted the post- survey, interview, and fMRI (when possible). Interviews were in-person or remote, via phone or WebEx, between August 2019-July 2020, lasted approximately 10 min, and were audio recorded. Participants were compensated $50 after the pre-study visit and another $75 after study completion.

### Design and Analysis

We used an open trial design with no control group to explore the feasibility and acceptability of this intervention. We collected qualitative data (via interviews) after the volunteering intervention and surveys before and after the volunteering intervention. Findings come from analyzing the open-ended interview data (see Appendix A for the interview guide) to explore themes related to participants’ volunteer experiences and perceptions of volunteering as associated with their mood and well-being. Interviews were transcribed verbatim and we used a matrix analysis approach (Averill, 2002) in which a study team member deductively constructed a matrix of interest areas (domains) to organize the data based on the interview guide and qualitative study aims. Data were summarized into key points and organized in the matrix, along with illustrative quotes. Participant responses were then synthesized into themes within each domain.

## Results

### Feasibility

Overall, providers were enthusiastic about the study; we met our recruitment target; and the intervention was acceptable for teens and providers. We describe two key challenges to feasibility, and the adaptations we made.

#### Key Challenge #1: Recruitment

The first key challenge to feasibility was finding clinical providers with enough clientele who met study inclusion criteria. We discussed this study with 22 clinical providers; conversations were extremely fruitful, and providers were enthusiastic about the project. Many saw the value of volunteering for some of their clients, and some already suggested volunteering with clients in therapy. Some lacked knowledge of local volunteer opportunities, so welcomed this support from our study. However, many clinical providers did not think they had enough clientele to refer to the project due to our initial criteria (only MDD single episode diagnoses and having been in treatment for less than 3 months). To address this, we (1) expanded our inclusion criteria to include mild/moderate anxiety, single episode (unspecified; separation; other specified) and adjustment disorder (unspecified; with depressed mood; with anxiety; with anxiety and depressed mood) in addition to MDD (unspecified; mild; moderate; partial remission; unspecified depressive disorder) and (2) expanded to include teens who had been in treatment up to 8 months. Once these modifications in criteria were made, we were able to recruit teens fairly seamlessly. Four referring providers had 16 conversations about the study with eligible clients. Of those, 12 were interested in participating in the study. Of the 12, we successfully recruited nine into our study. Eight out of nine completed the study, and one was lost to follow-up. Four of the nine completed their 30 volunteering hours; several had to stop early due to COVID-19 (Table 2).

**TABLE 2:** Summary of study participants’ volunteer experience.

Volunteering	*N* (out of *N* = 9)
**Volunteering experience before study**	
None	1
Occasional	4
Regular	2
Extensive	1
**Organizations chosen for study volunteering**	
Humane society	3
Church	2
Foodbank	1
School-based clubs	1
Theater festival	1
**Volunteer hours during study**	
<20	4
21–35	3
>35	2
Volunteering affected by COVID-19? (Yes/No)	5 (3)

#### Key Challenge #2: COVID-19 Challenges

A second key challenge to this study was disruption due to COVID-19. Three study participants completed the study before COVID-19 (before March, 2020); five study participants had to pause their volunteering (although two had already completed 30 h). We paused data collection completely for 3 months and eventually transitioned to virtual data collection for the post survey and interviews. The months-long delay made it unlikely that we would see any Default Mode Network activity corresponding to the volunteering intervention; we stopped post fMRI data collection for six of our nine participants. Some post data were collected virtually in the summer of 2020; we asked about life during COVID-19 during interviews and had a sense that it was difficult for teens and that most had developed ways to cope.

### Experiences With Volunteering

Most teens had occasional volunteer experience prior to study participation. Teens tended to choose to volunteer at organizations such as the Humane Society and local churches or schools (Table 2). Qualitative interviews with participants indicated that teens were highly favorable about their volunteer experiences, and all expressed an interest in continuing to volunteer following the study (see Table 3 for themes and illustrative quotes). An important aspect of enjoyment was the *alignment of the volunteer organization and/or specific duties with the individual’s interests*. Several participants shared that they specifically enjoyed the opportunity to work with children or animals, or that their volunteer experience aligned with their career interests. Participants also discussed the importance of having *choice* about where they volunteered, which some noted was different from past volunteer experiences. Finally, participants noted when the volunteer experience was *rewarding* suggesting that volunteering can provide satisfaction and enjoyment derived from helping others.

**TABLE 3:** Qualitative data domains, themes, and illustrative quotes.

Domain	Theme	Illustrative quote
Experiences with volunteering	Alignment with interests	*“I helped out in children’s church. I was a helper. Since I wanna work in pediatrics, that’s really helpful—‘cause I like kids in general.” (Participant 005)*
	Choice	*“Usually with [previous volunteer experience], it was just something that—it was just like whatever was available. It wasn’t something that I really chose, and I chose to volunteer at the Humane Society.” (Participant 007)*.
	Rewarding	*“I liked working with the other people ‘cause they don’t have many volunteers to help them out*…*That was enjoyable ‘cause knowing that I could help them out so they wouldn’t have to stress as much.” (Participant 010)*
Psychosocial assets	Feeling of giving back	*“You think about what this is gonna do for someone in the long term. With the food drive, we usually feed over 2,000 people with the food that we have because a lot of people don’t eat. That’s keepin’ people from bein’ hungry. That’s fillin’ people’s stomachs. You think to yourself, ‘I never really had to deal with that.’ The fact that we’re helping other people in the process, so they don’t have to go home and be hungry. It’s just crazy. We can put clothes on people’s backs over stuff that we don’t wear anymore. It’s like, ‘Wow. We’re really making a difference for someone.” (Participant 005)*
	Skills and opportunities	*“It’s a great learning opportunity. Plus, if you really want to get into college, then you need to. It’s good to do to a community as well.” (Participant 003)*
	Expanded worldview	*“After the day from the National Black Theatre Festival, I was pretty happy ‘cause people were complimenting me. No one was being racist to me, which I was happy about ‘cause I thought I was gonna get yelled at for being a white person in a black cultural event. They were all pretty nice*…*“I thought there would be one or two other white people, or there wouldn’t be a lot of Asians or stuff, but there was quite a few” (Participant 002)*
	Sense of purpose	*“It helps you get out of bed and also doing something productive, so it helps with depression and stuff ‘cause you’re actually doing something and seeing other people. Interacting with other people.” (Participant 010)*
	Acceptance	*“You shouldn’t be worried about it because it’s just kids. The kids are gonna love you because it’s you*…*You don’t have to be fake or pretend, just be yourself, and they’re gonna love you.” (Participant 008)*
	Challenges in volunteer experiences	*“I enjoyed helping people, but the only thing was I didn’t enjoy packing the pallets for the State because I’m not very strong, and they were very big pallets.” (Participant 008)*
Volunteering and mental health	Positive mood and well-being	*“My mom noticed that when I volunteer at the Humane Society, when I’m done, she always sees my happy smiles, and then I’m like- I just look like I feel so much better*… *I’ve noticed that service really makes- it makes people happy more. It’s always good to help you. Volunteering makes your mood good. If you have an emotional disorder, I think it will help” (Participant 001).*
	Social anxiety	*“I think I am learning to be a little more interacting with people. I’m not the type of person who would just have a conversation with you. They would have to talk to me first before I talked to them*…*I had to talk when I volunteered. I had to push myself out there.” (Participant 008).*
	Shift in focus, positive distraction	*“It distracts you from being anxious or being sad. You’re just happy. That’s the kind of feelin’ I want for someone with depression to have. I don’t want them to be sad. I don’t want to be sad. [Volunteering] makes me happy. I don’t feel anxious when I’m around them or doing things that help other people.” (Participant 005)*
	Challenges to mental health	*“I guess it was when there wasn’t a lot of volunteers. There were more kids than volunteers, was challenging. [Interviewer: What was that like?] It was really hectic. [Interviewer: How did you feel on those days when it was really hectic?]. I guess I sometimes felt overwhelmed.” (Participant 009)*

### Psychosocial Assets

Participant interviews revealed several psychosocial assets that were bolstered through volunteering and also noted challenges (Table 3). A positive aspect of the volunteer experience for adolescents and young adults was the *feeling of giving back/making a difference*. Several participants shared that they enjoyed “helping people,” or “making a difference for someone.” In addition to such psychological benefits, participants noted practical benefits such as gaining *skills and opportunities* and would recommend volunteering to other teens for opportunity to learn new things and for instrumental benefit such as building a resume or getting into college. One participant discussed how her volunteer experience challenged her and *expanded her worldview*, in particular, her expectations about racism and inclusivity. One participant explained how volunteering helped with her depression because it gave a *sense of purpose* and one participant recommended specifically that adolescents and young adults with anxiety should volunteer with children because children *accept you as you are*. When asked what was challenging, most participants shared *challenges* that they considered to be minor such as physical demands and getting children to behave.

### Volunteering and Mental Health

Participants were asked about how their volunteering related to their mood and well-being, and if they would recommend volunteering for other adolescents and young adults with anxiety and/or depression (Table 4). All participants felt that volunteering *positively affected their mood and well-being*. Some felt happier when they volunteered, and one described her volunteer experience as “relaxing” and “calming.” Several participants shared that volunteering helped with their *social anxiety*, and that it was beneficial to push themselves to talk to people as part of their volunteer experience. All participants said that they would recommend volunteering to other adolescents and young adults with anxiety and/or depression. Several participants also described the benefit of volunteering in terms of *shifting their focus and providing positive distraction*. One participant felt that volunteering was different from other activities (e.g., music) because it is “like a job.” Two participants specified that it can get someone’s mind off of their anxiety and/or depression. Participants also described two types of *challenges*: volunteering provoking anxiety and motivational challenges. Two participants described feeling “overwhelmed” or experiencing anxiety-provoking moments during volunteering even though both participants were positive about their overall experience. Participants also shared that it was sometimes hard to be motivated to go to their volunteer assignment, but that they felt better while they were volunteering or afterward. It is noteworthy that participants discussed overcoming challenges during their volunteering.

**TABLE 4:** Mental health before and after volunteering.

Mental health outcome	Mean before volunteering	(sd)	Mean after volunteering	(sd)	Min	Max
**Depressive symptoms**	21.222	(9.13)	12.625	(4.75)	3.00	31.00
* **Anxiety**						
Irritability	0.667	(0.87)	0.5	(0.53)	0.00	2.00
Agitation	1.11	(0.60)	0.375	(0.52)	0.00	2.00
Sleep disturbance (changes in sleep pattern)	1.889	(1.05)	0.875	(0.64)	0.00	3.00
Concentration difficulty	1.33	(0.71)	0.75	(0.71)	0.00	2.00

*Anxiety items are from the BDI (the four items are also included in the overall depression score). These items are pulled out to specifically examine an outcome related to anxiety.

On average, participants reported a 19% decrease in depressive symptoms from before to after volunteering, calculated as average relative change scores on the BDI from Pre- to Post-. Participant BDI scores were 21.11 on average (Pre) to 12.63 (Post); this is a clinically significant change from the “moderate” to the “minimal” range for BDI scores. Decreases were observed on the four BDI items relevant to anxiety (Table 4).

## Discussion

Despite potential motivational and logistical challenges [see Ballard et al. (2021)] for a discussion, teens who were referred to our study were very interested in participating. Once enrolled, they were able to find meaningful volunteer opportunities and complete their hours (with the exception of disruptions due to COVID-19). Several factors made this feasible. Clinicians had a good sense of their clients’ readiness, which teens would benefit, and which families would be able to facilitate community volunteerism. In addition, the study set up an infrastructure to help participants find a local opportunity corresponded with volunteer organizations to aid teens in completing paperwork as needed and texted participants every few weeks to check in. This infrastructure would be hard for clinicians to provide so it is worth understanding the role this support played and additional ways to minimize clinician burden.

Participants’ experiences in volunteering were overwhelmingly positive. All participants planned to volunteer following the study. Specifically, results points to the importance of volunteer experiences fitting with teens’ interests and having the ability to choose their volunteer opportunity. Participants also emphasized that it was rewarding to help others.

Regarding psychosocial assets by which volunteering can decrease depressive symptoms, interviewees discussed the importance of feeling like they were making a difference via volunteering, developing tangible skills, and feeling accepted as well as a gaining a sense of purpose. This aligns with and extends previous research suggesting that volunteering can help develop independence and personal worth, social connectedness, sense of purpose, practice with problem-solving and leadership skills, increase the ability to work with others, and develop particular skill sets, which can contribute to positive mental health benefits [e.g., Malin et al. (2015), Piliavin and Siegl (2015), and Creaven et al. (2017)]. These are potentially important clinical targets for teens struggling with depression and anxiety.

Regarding changes in mental health outcomes before and after the volunteering intervention; participants discussed many ways that volunteering affected their mood and well-being. Specifically, volunteering made some feel happy and calm, served as a positive distraction for others, and decreased their social anxiety by giving them a structured opportunity to interact with others. A notable (although preliminary) finding was the decrease in depressive symptoms from the clinically “moderate range” to “minimal” range. Although we did not test statistical significance, this is a clinically significant decrease in depressive symptoms, and the 19% decrease is above what is considered a minimally clinically significant change [e.g., 17.5%, Button et al. (2015)]. Given the study design, we cannot attribute this decrease in depression severity to volunteering alone; participants continued with their treatment as usual over the time-frame of the study as volunteer experiences were intended to be a supplemental intervention to primary treatment.

### Limitations and Next Steps

This exploratory feasibility study did not include a control group which makes it impossible to determine the unique benefits and additive effects of community volunteering for developmental or treatment-as-usual effects. The small sample size, although by design, is a limitation regarding statistical significance testing and was not diverse with regards to gender or race/ethnicity, which limits generalizability. This exploratory study provides a basis for future research using rigorous design to compare treatment-as-usual to treatment-as-usual plus volunteering with a larger sample. Future studies should also include an explicit measure of anxiety to better assess the role of volunteering in reducing anxiety symptoms. Finally, future studies with larger samples can continue to test which aspects of volunteering are most beneficial for teens in the treatment of depression and anxiety, how to make such opportunities more broadly available to all teens, what supports make this intervention feasible for provides, and what is the necessary “dose” of volunteering to provide benefits to teens.

### Implications

Our preliminary findings suggest that clinical providers treating adolescents and young adults for mild to moderate depression and/or anxiety may wish to consider incorporating community volunteer activities into treatment. Community volunteering was desirable and feasible participants. Incorporating volunteerism may be a useful modality in the treatment of internalizing disorders to increase behavioral activation, socialization, participation in meaningful activities, a focus on others rather than self, self-esteem and self-efficacy, and routine and structure. When considering who might benefit most, clinicians would need to consider diagnoses, disorder severity, and course of treatment. Clinicians might search for existing local resources that compile teen-friendly volunteering opportunities and consider how best to partner with local organizations to support alignment between youth volunteers and organizations. In sum, the use of volunteering as an addition to standard therapy approaches has promise for the treatment of depression and anxiety for adolescents and young adults, this strategy merits further empirical investigation.

## Data Availability Statement

The raw data supporting the conclusions of this article will be made available by the authors, without undue reservation.

## Ethics Statement

The studies involving human participants were reviewed and approved by Wake Forest Institutional Review Board. Written informed consent to participate in this study was provided by the participants’ legal guardian/next of kin.

## Author Contributions

PB conceptualized and led the writing for this manuscript and conducted the quantitative analysis. SD contributed to the conceptualization and writing. GA contributed to the literature review. AS conducted the qualitative analysis. SD, GA, AK, LN, EC, and AS contributed to discussing, framing, reviewing the literature, and editing the manuscript. All authors contributed to the article and approved the submitted version.

## Conflict of Interest

The authors declare that the research was conducted in the absence of any commercial or financial relationships that could be construed as a potential conflict of interest.

## Publisher’s Note

All claims expressed in this article are solely those of the authors and do not necessarily represent those of their affiliated organizations, or those of the publisher, the editors and the reviewers. Any product that may be evaluated in this article, or claim that may be made by its manufacturer, is not guaranteed or endorsed by the publisher.

## Footnotes

1 Originally, this study included an exploratory aim to investigate a potential neurobiological mechanism (activity in the Default Mode Network) linking volunteering and affective disorders. We completed nine pre fMRI visits with participants. To comply with safety protocols during the COVID-19 pandemic, we stopped in person fMRI visits and had only completed two post fMRI visits. Thus, fMRI data is not included in this study. Please email the first author for further detail about this component of the study.

## Appendix

**Appendix A|** Interview Guide.

Thank you for taking part in this study!

Can you tell me about your experience with volunteering?

  *Probe: What did you enjoy? What was challenging? How many hours did you complete?*

How did Covid-19 (coronavirus) affect your volunteering for this study?

Do you think the volunteering affected your mood or well-being? How so?

Do you think you will continue volunteering or stop? Can you tell me why?

What was it like completing surveys? How about doing fMRI scans?

Would you recommend volunteering to other teens? Why or why not?

How has Covid-19 (coronavirus) affected your mood or well-being?

How has Covid-19 (coronavirus) affected your feelings of connection to other people and your community?

Do you have any questions for me?

Again, thank you for taking your time to speak with me and to participate in this research.
